# A Literature Systematic Review with Meta-Analysis of Symptoms Prevalence in Covid-19: the Relevance of Olfactory Symptoms in Infection Not Requiring Hospitalization

**DOI:** 10.1007/s11940-020-00641-5

**Published:** 2020-08-28

**Authors:** A. Giorli, F. Ferretti, C. Biagini, L. Salerni, I. Bindi, S. Dasgupta, A. Pozza, G. Gualtieri, R. Gusinu, A. Coluccia, Marco Mandalà

**Affiliations:** 1 Otolaryngology Department, Azienda Ospedaliera Universitaria Senese, Siena, Italy; 2 Dipartimento Scienze Mediche Chirurgiche e Neuroscienze, Università di Siena, Siena, Italy; 3 Department of Audiovestibular Medicine and Neurotology, Alder Hey Children’s Hospital NHS Trust, Liverpool, UK; 4 United Kingdom and Sheffield Vertigo and Balance Centre, Sheffield, UK

**Keywords:** COVID-19, Sars-CoV-2, Olfactory disorders, Meta-analysis, Hospitalization

## Abstract

**Purpose of review:**

To investigate the association between the olfactory dysfunction and the more typical symptoms (fever, cough, dyspnoea) within the Sars-CoV-2 infection (COVID-19) in hospitalized and non-hospitalized patients.

**Recent findings:**

PubMed, Scopus and Web of Science databases were reviewed from May 5, 2020, to June 1, 2020. Inclusion criteria included English, French, German, Spanish or Italian language studies containing original data related to COVID19, anosmia, fever, cough, and dyspnoea, in both hospital and non-hospital settings. Two investigators independently reviewed all manuscripts and performed quality assessment and quantitative meta-analysis using validated tools. A third author arbitrated full-text disagreements. Following the Preferred Reporting Items for Systematic Reviews and Meta-Analyses (PRISMA), 11 of 135 studies fulfilled eligibility. Anosmia was estimated less prevalent than fever and cough (respectively rate difference = − 0.316, 95% CI: − 0.574 to − 0.058, *Z* = − 2.404, *p* < 0.016, *k* = 11 and rate difference = − 0.249, 95% CI: − 0.402 to − 0.096, *Z* = − 3.185, *p* < 0.001, *k* = 11); the analysis between anosmia and dyspnoea was not significant (rate difference = − 0.008, 95% CI: − 0.166 to 0.150, Z = − 0.099, *p* < 0.921, *k* = 8). The typical symptoms were significantly more frequent than anosmia in hospitalized more critical patients than in non-hospitalized ones (respectively [*Q*(1) = 50.638 *p* < 0.000, *Q*(1) = 52.520 *p* < 0.000, *Q*(1) = 100.734 *p* < 0.000).

**Summary:**

Patient with new onset olfactory dysfunction should be investigated for COVID-19. Anosmia is more frequent in non-hospitalized COVID-19 patients than in hospitalized ones.

## Introduction

In Wuhan, China, in December 2019, a previously unidentified coronavirus emerged and spread to many other Chinese cities and then globally [1]. On March 11, 2020, the World Health Organization declared the coronavirus disease 2019 (COVID-19) outbreak as a pandemic [2].

The virus responsible for the pandemic is known as a novel coronavirus, which can generate a severe acute respiratory syndrome and was named SARS-CoV-2. In the past 20 years, two other new coronaviruses, the SARS-CoV (2003) and the MERS-CoV (2012), had emerged. The typical clinical manifestations for these virus infections were fever and respiratory symptoms with different percentages of mortality [1].

Indeed, the clinical characteristics of the SARS-CoV-2 typify respiratory viruses, fever, cough and shortness of breath, are the most important symptoms. These generally lead to testing, and then a diagnosis of COVID-19 can be confirmed or excluded. Gastrointestinal symptoms may be present as well as symptoms of the upper airways, but they are by no means characteristic of the disease and their prevalence is lower. Radiological findings may show a bilateral pneumonia with ground-glass opacity. When developing an acute respiratory distress syndrome, patients can worsen in a short time and then can die of multiple organ failure [3]. In particular, people older than 70 are specially at risk due to rapid progression of the disease and death. Consequently, the highest rate of deaths is observed in elderly people [4].

As a new viral infection, it is not known exactly how the COVID-19 virus can affect general health. Therefore, improved understanding about its natural history is important that has profound implications on a timely diagnosis and in taking public health measures to prevent the spread of the virus [5].

The potential capability of asymptomatic patients to spread the infection was considered since the early stages of the pandemic [6]. This evidence grew stronger while the pandemic spread faster all over the world, emphasizing the importance of the screening and the detection of asymptomatic cases, as they may be excluded from public health strategies and may continue to spread if not identified at the right time [7, 8]. Identifying and isolating paucisymptomatic patients (those without the so-called typical symptoms) therefore becomes crucial to prevent further outbreak of the disease and to contain the load in our healthcare systems [9].

SARS-CoV-2 enters the host through the mucosa of the respiratory tract or other mucosal surfaces (conjunctiva) [1]. The major site of concentration determining the infection is the nasopharynx [10].

SARS-Cov-2 has been shown to have a human-to-human transmission [11, 12] with a basic reproduction number (R0) ranges from 2.24 and 3.58 at the beginning of the pandemic [13], decreased only by preventing measures of isolation enforced by public health interventions [14].

SARS-CoV-2 is a novel bat-derived coronavirus that enters the human cells using a spike protein (S) and that binds the ACE2 protein on target cells [15], after its cleavage by cell surface protease such as TMPRSS2.

It has been described that ACE2 and the TMPRSS2 are expressed in the human olfactory epithelium support and stem cells, as well as the vascular pericytes in the nose and in the olfactory bulb. They are not directly expressed by the neurons in the olfactory bulb [16]. So, the damage to the olfactory system could be a result from the local infection of support cells, altering temporarily the signalling from the olfactory neurons and bulb, or damage to the entire structure of the olfactory epithelium, causing a permanent dysfunction of the olfactory pathway [16]. Furthermore, SARS-CoV-2 seems to involve the nervous system, and the neurological manifestations seem to be more evident in patients with severe symptoms [17, 18]. On the other hand, several viruses are well-known to cause post-viral olfactory dysfunction, for example, rhinoviruses as well as coronaviruses, parainfluenza and Epstein-Barr virus [19]. This is not unsurprising as these viruses colonize the upper airway tracts.

Since the first report from ENT UK who drew attention to the observation that an increasing number of patients showing anosmia without other symptoms were found out to be positive with SARS-CoV-2 infection [20], many investigations are ongoing to understand the role of this particular symptom [21–25]. Thus, it will be rational to think that olfactory symptoms may be typical symptoms of SARS-CoV-2 in addition to the traditional ones of fever, cough and respiratory distress.

Our objective is to observe whether there is any significant difference in the relative prevalence of anosmia when compared with the more traditional symptoms of COVID-19. This will lead to better understanding of anosmia as a symptomatic marker of the infection, especially in paucisymptomatic patients. To achieve this, we performed a meta-analysis of the evidence in published literature to investigate the association between the presence of anosmia/ageusia and the symptoms known as “typical” for COVID-19 (fever, cough, dyspnoea).

## Methods

### Design

In this meta-analysis, our search was performed following the Preferred Reporting Items for Systematic Reviews and Meta-Analyses (PRISMA) [26]. Due to this research method, the registration with our institutional review board was not required.

### Eligibility criteria

For the present review, we searched for articles aiming to explore the prevalence of anosmia, fever, cough and dyspnoea in a population of COVID-19-positive patients either in the hospital or outside. Studies were included if they met the following criteria (Table 1): (a) they reported data necessary to calculate the fixed or random effects pertaining to the prevalence of the symptoms of anosmia, fever, cough and dyspnoea; (b) total sample sizes and the number of patients presenting the different symptoms were of enough power adequate; and (c) the paper was published in English, French, German, Spanish or Italian language. Reviews, single-case studies, case series and case reports were not considered. Cross-sectional, case-control or retrospective observational studies were considered eligible designs. Longitudinal studies were included if they reported a baseline data regarding the prevalence of symptoms in this type of population or the authors were available to provide them if requested.

**Table 1:** Descriptive characteristics of the included studies (*n* = 11).

First author and year	Country	Study design	Recruitment setting	Total no.	Mean or median age (year)	Covid-19 testing	Mode of testing anosmia
Aggarwal et al. 2020 [27]	USA	Retrospective observational	Inpatients	16	Median (67, 0)	RT-PCR	Clinical records
Giacomelli et al. 2020 [25]	Italy	Cross-sectional	Inpatients	59	Median (60, 0)	Not reported	Self-report survey
Klopfenstein et al. 2020 [28]	France	Retrospective observational	Inpatients and outpatients	114	Mean (47, 0)	RT-PCR	Clinical records
Lechien et al. 2020 [29]	France, Italy, Spain, Switzerland	Cross-sectional	Inpatients	1420	Mean (39, 2)	RT-PCR	Physical exam
Levinson et al. 2020 [30]	Israel	Cross-sectional	Inpatients	42	Not reported	RT-PCR	Self-report survey
Moein et al. 2020 [31]	Iran	Case control	Inpatients	60	Mean (46, 6)	RT-PCR	Physical exam
Tostmann et al. 2020 [32]	Netherlands	Cross-sectional	Healthcare workers	79	Not reported	Not reported	Self-report survey
Vaira et al. 2020 [33]	Italy	Cross-sectional	Inpatients	72	Mean (42, 9)	Not reported	Clinical records
Wee et al. 2020 [34]	Singapore	Cross-sectional	Inpatients and outpatients	35	Not reported	RT-PCR	Self-report survey
Yan et al. 2020 [35]	USA	Retrospective observational	Inpatients and outpatients	128	Median (admitted = 53, 5; ambulatory = 43, 0)	PCR	Clinical records
Yan et al. 2020 [36]	USA	Cross-sectional	Inpatients and outpatients	59	Not reported	PCR	Self-report survey

### Information sources and search procedure

Our search strategy used the PubMed (via the web), Scopus and Web of Science databases, and it started on May 5, 2020, using keywords, and where possible Mesh terms, related to *COVID-19*, *anosmia*, *fever*, *cough* and *dyspnoea*. In addition, hand-searched articles unavailable in the cited databases were identified and also included. The search was restricted to those papers published after the year 2019.

### Selection of studies

Two of the authors (AP, FF) independently screened selected articles in three phases. During the first and second stages, studies were examined with regard to the inclusion criteria after reading the title and the abstract, respectively. If the contents of the title or abstract were unclear, or if there was disagreement between the authors on inclusion or exclusion, the article was selected for the following stage. During the final stage, two authors examined independently the full text of the papers. Through consensus of the third reviewer (AC), full-text disagreements were resolved. Only peer reviewed full-text publications where considered in this selection phase. Articles were included only if the articles reported on the prevalence of anosmia, fever, cough and dyspnoea. Studies concerning animal or laboratory data, review/meta-analyses, case report and duplicate data were excluded. There was no disagreement during the article selection process. Finally, two other co-authors (SDG and MM) critically reviewed the selection process.

**Fig. 1:**
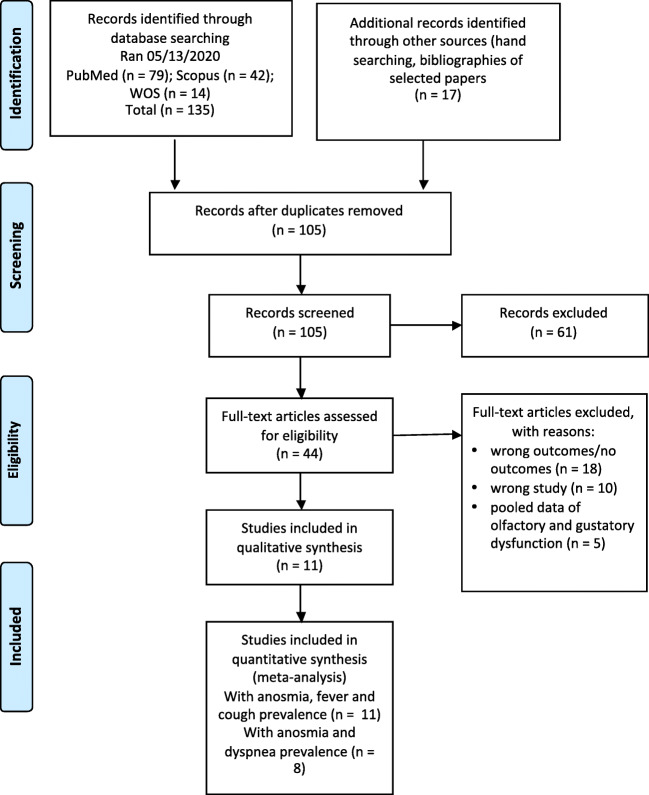
PRISMA flowchart of the study selection.

The PRISMA flowchart of the study selection process is provided in (Fig. 1). Our research strategy provided 135 results from the databases and 17 papers via hand searching. After removing the duplicates, 105 articles were screened and 61 were excluded. Forty-four articles underwent full-text review, resulting in the exclusion of 33 studies and yielding a total of 11 articles included in the analysis. Eight out of 11 studies provided the prevalence of anosmia, fever and cough symptoms, but not the prevalence of dyspnoea.

### Data extraction and coding

Two authors (AP, FF) reviewed the 11 studies included in the data extraction process, and the third author (GG) was consulted to resolve disagreements.

Data were inserted into an Excel worksheet, and the following information were extracted and coded from each study: (1) title of the paper, (2) first author, (3) publication date, (4) country where the study was conducted, (5) research design, (6) recruitment setting, (7) total sample size, (8) mean/median age, (9) COVID-19 testing, (10) mean age of the offspring total group, (11) mode of testing anosmia, (12) number of patients with anosmia and its prevalence, (13) number of patients with fever and its prevalence, (14) number of patients with cough and its prevalence and (12) number of patients with dyspnoea and its prevalence. After entering the data, any discrepancies were discussed at a meeting between the authors who extracted the data and the third author.

### Statistical analysis

Statistical analyses were performed using CMA v3. Considering that prevalence could be affected by the characteristics of the populations included, and considering the heterogeneity of the methods used to assess olfactory dysfunctions, we used random-effects models which allow the true effect sizes to differ from study to study [37]. Three meta-analyses were computed, with the aim of comparing anosmia against the other symptoms (fever, cough and dyspnoea). The effect sizes were calculated as the difference between the prevalence of anosmia vs fever, anosmia vs cough and anosmia vs dyspnoea. Positive effect sizes indicated that the difference was in favour of anosmia. The effect sizes were estimated by adopting a 95% confidence interval computed for a proportion. Forest plots were created, and heterogeneity analysis of the effect sizes was performed by calculating the Higgins’s *I*^2^ statistic [38] and the Cochrane’s *Q* index [39]. A Cochrane’s *Q P* value < 0.1 and an *I*^2^ > 40% were considered markers of heterogeneity.

Publication bias was explored through the inspection of the funnel plot and the Egger’s test [40]. The funnel plot appears asymmetrical if publication bias is detected, while a non-statistically significant result of the *t* value of the Egger’s regression intercept allows us to reject publication bias.

Three sensitivity analyses were performed, for each symptom’s comparisons with anosmia (fever, cough, dyspnoea). The effect sizes were computed in the studies including only inpatients or in those which enrolled mixed populations (inpatients and outpatients).

## Results

### Descriptive characteristics of the studies

The sample sizes in the included studies ranged from 16 to 1420 participants. Three studies were conducted in the USA, one in Israel, one in France, two in Italy, one in Singapore, one in the Netherlands, one in Iran and one study recruited patients in four countries (France, Italy, Spain, Switzerland). One study was conducted on healthcare workers, 6 on inpatients and 4 studies both inpatients and outpatients. Among the selected studies, we included only one case control, 7 were cross-sectional and 3 retrospective observational ones. A large heterogeneity was found about the mode of testing anosmia: 5 studies used a self-report survey, 4 used clinical records and 2 obtained data through physical exams. The descriptive characteristics of the included studies are presented in Table 1.

### Anosmia vs fever

**Fig. 2:**
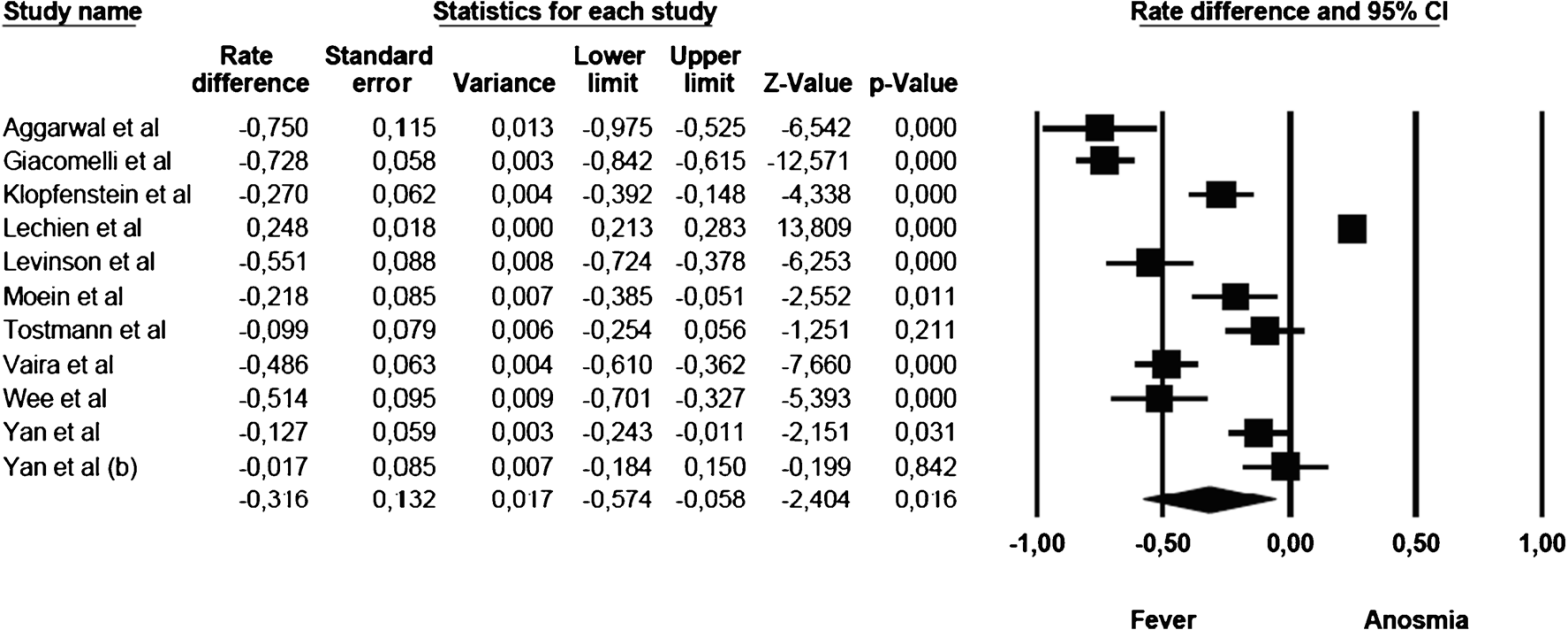
Forest plot of effect sizes: rate difference of anosmia vs fever.

The mean effect size was statistically significant and showed that the commoner prevalence was fever (rate difference = − 0.316, 95% CI: − 0.574 to − 0.058, *Z* = − 2.404, *p* < 0.016, *k* = 11). The forest plot with mean effect sizes is provided in Fig. 2. For this analysis, a significant heterogeneity was found [*I*^2^ = 98.176, *Q*_(10)_ = 67.906, *p* < 0.000]. Funnel’s plot examination showed an asymmetric distribution of the studies: only 2 of them were located inside the funnel, while 8 out of 11 studies were asymmetrically distributed on the left side, but out of the funnel. It was observed that there was a publication bias as confirmed by the significance of the Egger’s test of intercept [*β* = − 10.106, SE = 2.143, *t*_(9)_ = 4.716, *p* < 0.001].

### Anosmia vs cough

**Fig. 3:**
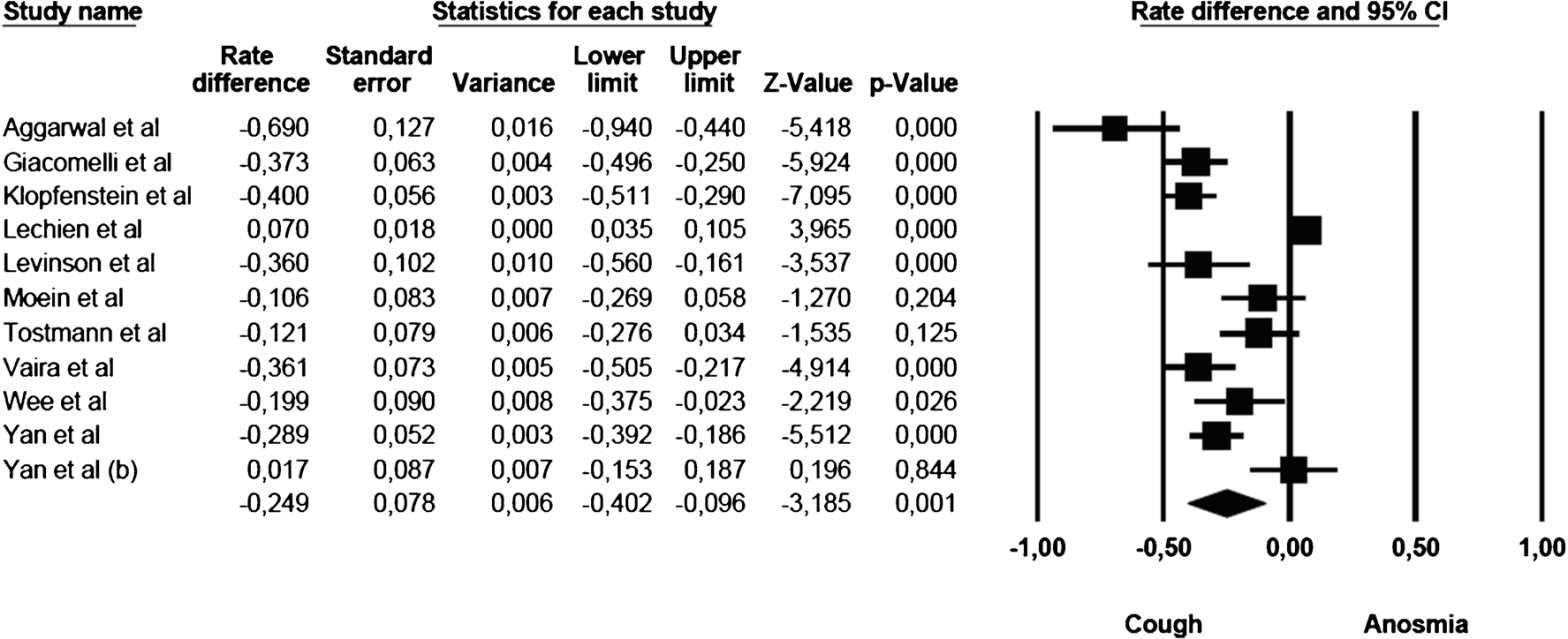
Forest plot of effect sizes: rate difference of anosmia vs cough.

The comparison of anosmia and cough prevalence showed that cough was commoner than anosmia. The mean effect size was statistically significant (rate difference = − 0.249, 95% CI: − 0.402 to − 0.096, *Z* = − 3.185, *p* < 0.001, *k* = 11). The forest plot with mean effect sizes is provided in Fig. 3. A significant heterogeneity was found [*I*^2^ = 94.693, *Q*_(10)_ = 188.435, *p* < 0.000], and publication bias was suggested by the Funnel plot (6 out of 11 studies were located on the left side out of the funnel) and by the significance of the Egger’s test of intercept [*β* = − 5.512, SE = 1.366, *t*_(9)_ = 4.035, *p* < 0.003].

### Anosmia vs dyspnoea

**Fig. 4:**
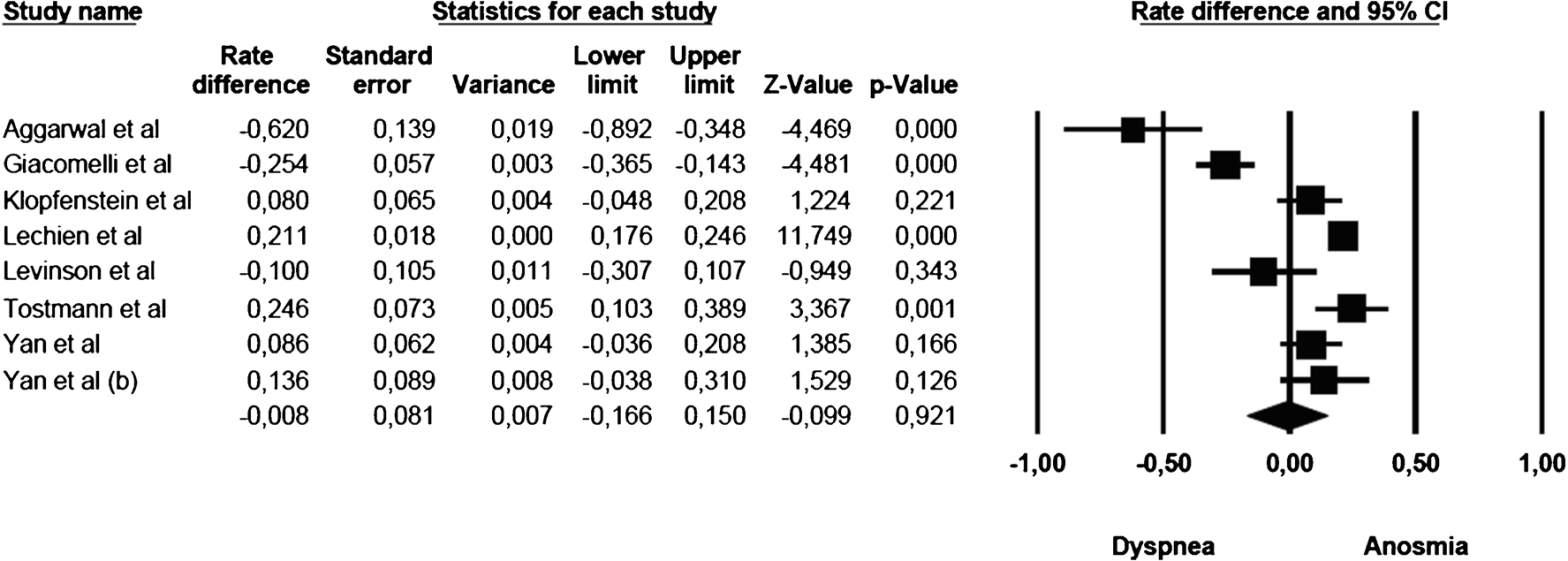
Forest plot of effect sizes: rate difference of anosmia vs dyspnoea.

The mean effect size was not significant (rate difference = − 0.008, 95% CI: − 0.166–0.150, *Z* = − 0.099, *p* < 0.921, *k* = 8): five studies showed a commoner prevalence of anosmia, while three studies showed a commoner prevalence of dyspnoea. The forest plot with mean effect sizes is provided in Fig. 4. A significant heterogeneity was detected for this analysis as well [*I*^2^ = 93.183, *Q*_(7)_ = 102.678, *p* < 0.000]. There was no evidence of publication bias, as suggested by the funnel plot (only 2 out of 8 studies were distributed on the left side out of the funnel) and by the Egger’s test of intercept which was not statistically significant [*β* = − 3.908, SE = 1.731, *t*_(6)_ = 2.257, *p* < 0.065].

### Sensitivity analysis

**Fig. 5:**
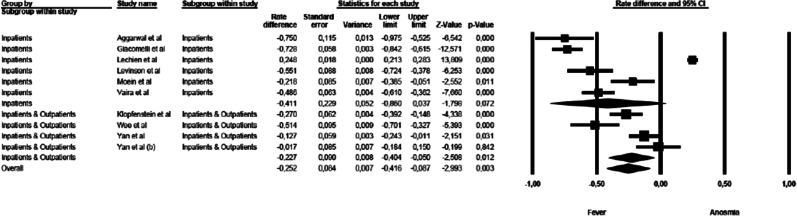
Forest plot of effect sizes across studies enrolling inpatients only and studies which used mixed samples (inpatients and outpatients)—anosmia vs fever.

The first sensitivity analysis was related to the symptoms of anosmia and fever (Fig. 5). A significant difference was detected in the mean effect sizes between the studies on inpatients and those on mixed populations [*Q*_(1)_ = 50.638, *p* < 0.000]. The symptoms of fever were more prevalent than symptoms of anosmia among studies conducted on inpatients, while these differences were milder in studies enrolling mixed populations.

**Fig. 6:**
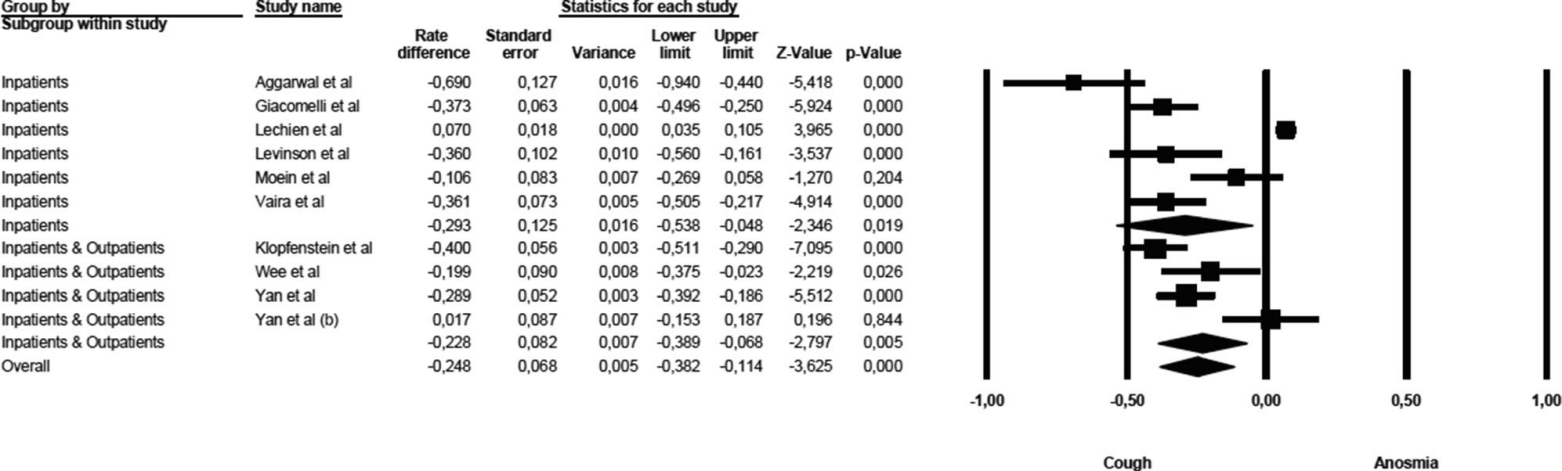
Forest plot of effect sizes across studies enrolling inpatients only and studies which used mixed samples (inpatients and outpatients)—anosmia vs cough.

A significant difference in the mean effect sizes was found for the comparison of anosmia and cough (Fig. 6), between the studies on inpatients and those on mixed populations [*Q*_(1)_ = 52.520, *p* < 0.000]: symptoms of cough are more prevalent than anosmia in inpatient samples, and this gap is milder in mixed populations.

**Fig. 7:**
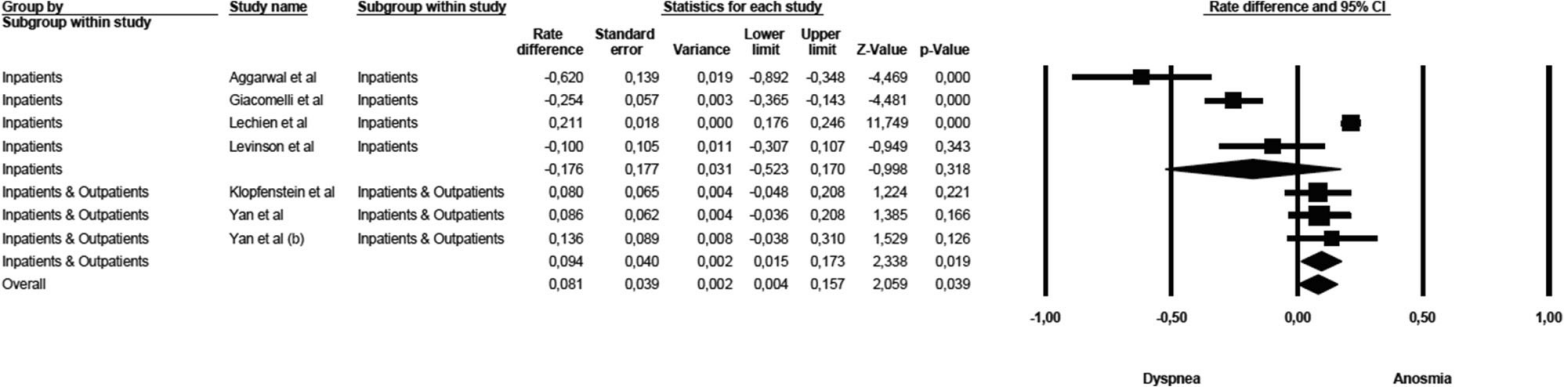
Forest plot of effect sizes across studies enrolling inpatients only and studies which used mixed samples (inpatients and outpatients)—anosmia vs dyspnoea.

In the sensitivity analysis, the symptoms of anosmia and dyspnoea were compared among studies including inpatients only and studies using mixed populations (Fig. 7). A significant difference was detected in the mean effect sizes between the studies [*Q*_(1)_ = 100.734, *p* < 0.000]. The symptoms of dyspnoea were more prevalent than of anosmia among studies conducted on inpatients, but symptoms of anosmia were more prevalent when mixed populations were considered.

## Discussion

Since the outbreak of COVID-19 pandemic, it has become clear that the virus affects the olfactory and gustatory senses.

The olfactory neurons, located in the olfactory epithelium in the so-called olfactory cleft, express circa specific 380 different receptors encoded by about 1000 different olfactory genes coded in our genome [41]. Different receptors cause corresponding different activations of the olfactory bulb, leading to a different signalling to the hippocampus, the amygdala and the orbitofrontal cortex. Neurological disorders such as Parkinson’s syndrome and Alzheimer’s disease are well-known to impair the olfactory capability [42]. Furthermore, olfactory neuroepithelium has also as immunosensory function elicited by the nitric oxide (NO) pathway that plays a role in arresting the spread of viral infection to the central nervous system, and clinically this activity corresponds to hyposmia or anosmia [43, 44].

Taste has 3 nervous pathways (the sensory branch of the intermediate nerve, the glossopharyngeal nerve and the vagus nerve). Trying to establish a relationship between an impairment of taste and one pathological condition, for example, the SARS-CoV-2 may be difficult due to this variability in taste perception [42]. In addition, many drugs can cause a modification of taste (e.g. clarithromycin and other antibiotics, corticosteroids; these two kinds of drugs are commonly given to COVID-19 patients, especially in the first weeks from the beginning of the infection) [45]. So, we felt that it was sensible to focus on the relationship between COVID-19 and olfactory disorders only.

A multicentric European study considered a population of 417 patients with mild-to-moderate COVID-19. In their experience, they found out that up to 85.6% of patients had olfactory dysfunction and 88.8% had gustatory disorders [22]. Olfactory disorders appeared before other symptoms in 11.8% of patients, an observation that might have important connotations for detecting patients in an early stage of infection, quarantining them and thereby preventing the spread of the disease. They considered both inpatients in hospitals and patients isolating in their homes.

All countries facing the emerging pandemic also had to contend with variable possibilities of self-hygiene and public health. So, it appears reasonable to consider the patients staying at home as patients with mild-to-moderate symptoms, while subjects hospitalized had more severe clinical manifestations.

The increase in the reporting of patients with a sudden onset of anosmia is supporting the evidence for this symptom as a specific clinical presentation of the SARS-CoV-2 infection. This is the Isolated Sudden-Onset Anosmia (ISOA) [23]. It is important to underline that patients affected with ISOA usually do not show any other rhinological symptom, such as rhinorrhea, or nasal obstruction. When patients are found with ISOA, they should be investigated for COVID-19 and isolated in the meantime [46]. In order to better understand the prevalence and the clinical importance of the symptoms and their progression related to the COVID-19 pandemic, the American Academy of Otolaryngology–Head and Neck Surgery (AAO-HNS) has established the COVID-19 Anosmia Reporting Tool for Clinicians [21]. This tool was created to allow healthcare providers of all specialties worldwide to submit data to describe their findings about clinical presentation of olfactory disorders in COVID-19 patients. They observed that anosmia occurred in 73% of patients before the diagnosis of SARS-CoV-2 infection. In more than one out of four of these patients (26.6%), anosmia was indeed the very first symptom they had. They also developed fever in 38% and cough in 41%. No data was available about dyspnea, but in this group, up to 27% did not develop any other symptom.

The present meta-analysis establishes that anosmia is a real and tangible symptom and deserves every possible consideration and importance when COVID-19 is suspected.

If we consider the subjective loss of smell, self-reported symptoms may be reported at a lesser rate than the validated symptoms [31]. This evidence may also depend on the fact that there is no data as to the presence of this self-reported symptom before it was identified. Inpatients seem to show a lower percentage of self-reported anosmia/hyposmia [25, 31].

Fever and cough are considered to be early symptoms for COVID-19 patients, and with dyspnoea, they are accepted as typical symptoms to identify potentially COVID-19 ill patients [4]. In other studies that included subjects with mild symptoms who tested positive for SARS-CoV-2, it was observed that anosmia was present in 46.8%. In these studies again, only 3.7% of patients who tested negative for SARS-CoV-2 showed anosmia. On the other hand, up to 58.9% of COVID-19 patients had cough, and 56.7% had fever, while shortness of breath was found in 22.2%, and they did not require hospitalization. In their experience, anosmia can be suggested as a strong predictor for COVID-19, with high sensitivity and moderate specificity [32].

The prevalence of anosmia in patients with severe acute respiratory syndrome caused by the SARS-CoV-2 with traditional symptoms was found out only in 19% of patients requiring hospitalization 50% of whom eventually required hospital admission [27].

The Italian experience in Lombardy also showed how only 33% of patients had impairment of the olfactory and gustatory function, while up to 72.8% presented with pneumonia at the time of hospital admission [25].

This meta-analysis demonstrates that anosmia although less prevalent than the traditionally accepted symptoms of cough and fever nevertheless is not uncommon and should be used as a warning symptom for further testing.

## Conclusions

Patients with sudden isolated olfactory dysfunction without any other symptoms except possible gustatory symptoms should be investigated for COVID-19. This may prevent the risk of spreading the disease from patients who are apparently healthy and therefore considered non-contagious. They might never develop traditional symptoms like cough, fever and dyspnoea. They may continue regular social and working activity, thereby increasing chances of spreading the infection to others. Isolating paucisymptomatic patients, that may well be in a greater percentage of infected people, will assist to contain the disease faster and in a more effective way. As our knowledge of the disease grows, we can recognize new emerging symptoms of the disease that can help us to trace and isolate more precisely. Recognizing COVID-19 in an anosmic patient with no other symptoms can help flatten the curve and a recurrence of the disease, guiding preventive measures.

## Limitations of the study

This meta-analysis has several limitations. The first limitation is a lack of homogeneity in providing a true prevalence of anosmia in COVID-19. Secondly, this symptom very often presented as “loss of smell”, and sometimes, it is pooled with data related to gustatory dysfunctions. Different methods of assessing anosmia were used in literature, and different groups of patients were recruited as well that is bound to raise confounding variables for meaningful deductions. All these limitations are probably responsible for the large heterogeneity revealed by the three meta-analyses showed in this paper. This issue deserves more attention in the future.
